# Dynamical diffraction of high-energy electrons by light-atom structures: a multiple forward scattering interpretation

**DOI:** 10.1107/S2053273322011779

**Published:** 2023-02-09

**Authors:** Tarik R. Drevon, David G. Waterman, Eugene Krissinel

**Affiliations:** a STFC, Rutherford Appleton Laboratory, Didcot, OX11 0FA, United Kingdom; b CCP4, Research Complex at Harwell, Rutherford Appleton Laboratory, Didcot, OX11 0FA, United Kingdom; Czech Academy of Sciences, Czech Republic

**Keywords:** high-energy electron diffraction, T-matrix, multiple scattering, independent atom approximation

## Abstract

Because of the strong electron–atom interaction, the kinematic theory of diffraction cannot be used to describe the scattering of electrons by an assembly of atoms due to the strong dynamical diffraction that needs to be taken into account. In this paper, the scattering of high-energy electrons by a regular array of light atoms is solved exactly by applying the T-matrix formalism to the corresponding Schrödinger’s equation in spherical coordinates. The independent atom model is used, where each atom is represented by a sphere with an effective constant potential. The validity of the forward scattering approximation and the phase grating approximation, assumed by the popular multislice method, is discussed, and an alternative interpretation of multiple scattering is proposed and compared with existing interpretations.

## Abstract 2

The T-matrix is used to compute the scattering of fast electrons by a regular array of effective spherical potential wells. An assessment of the forward scattering approximation and a real-space multiple scattering interpretation are provided.

## 1. Introduction

### 1.1. Motivation

The theory of dynamical diffraction was first developed in 1928 (Bethe, 1928 ▸; Cowley, 1995 ▸), very shortly after the first experimental demonstration of electron diffraction. Over the 20th century, several theories of electron diffraction have been extensively applied in various areas of solid-state physics. In particular, the multiple scattering theory (MST) (Korringa, 1947 ▸, 1994 ▸; Kohn & Rostoker, 1954 ▸; Dederichs, 1971 ▸) has proven to be very efficient for describing electronic properties of matter. Besides, MST provides an intuitive picture of dynamical diffraction. Partial wave scattering theory (Schiff, 1955 ▸) provides another description of multiple scattering with the possibility to take into account inelastic scattering (Howie, 1963 ▸) and loss of coherency (Howie, 2014 ▸). Other less commonly known attempts at an intuitive depiction of dynamical diffraction have been proposed to explain electron channelling in crystals, for example by VanDyck & Op de Beeck (1996 ▸). Dynamical diffraction must be taken into account when considering electron–atom interactions, since even at the very high electron energies commonly used in modern transmission electron microscopes, the interaction is so strong that, theoretically, the kinematic approximation is not valid for ideal crystals thicker than 10–15 nm (Hirsch *et al.*, 1965 ▸; Glaeser & Downing, 1993 ▸; Subramanian *et al.*, 2015 ▸). In practice, crystal growth cannot be controlled to such a degree of accuracy and, usually, nanocrystals of organic compounds have a size on the order of hundreds of nanometres. This is a challenging aspect of high-energy electron diffraction in crystallography, as it significantly complicates the structure determination process. However, structures have been successfully determined from ED (electron diffraction) data using the standard kinematic theory (Gemmi *et al.*, 2019 ▸; Nannenga *et al.*, 2014 ▸; Nannenga & Gonen, 2019 ▸). Although dynamical refinement (Palatinus *et al.*, 2013 ▸) usually leads to better intensity predictions (Gemmi *et al.*, 2019 ▸), the agreement between theory and experiment is still significantly worse than that obtained for X-ray data (Oleynikov *et al.*, 2007 ▸; Klar *et al.*, 2021 ▸).

Although there is no quantitative prediction of the effects of dynamical diffraction on the ability to solve a crystal structure, it may be speculated that dynamical diffraction may be attenuated by other effects such as a lack of coherency caused by solvent scattering or crystal defects. Therefore, more accurate theoretical models are needed for getting better results from electron diffraction experiments.

### 1.2. State of the art

The theory of multiple scattering has a long and rich history, including modern theoretical developments and implementations (Sébilleau *et al.*, 2017 ▸; Zabloudil *et al.*, 2005 ▸). However, in the context of high-energy electron diffraction, the multislice (MS) (Cowley & Moodie, 1957 ▸) approach and Bloch-wave (BW) (Bethe, 1928 ▸; Metherell & Fisher, 1969 ▸) approach are the most popular methods for simulating diffraction patterns by crystals.

MS is particularly well suited for solving large problems as it involves successive convolutions, which can be very efficiently computed with the fast Fourier transform (FFT) (Ishizuka & Uyeda, 1977 ▸). However, in order to avoid aliasing, transverse periodic boundary conditions must be met. This is not always possible if the crystal is in an arbitrary orientation as is the case in typical continuous-rotation electron diffraction experiments. A small beam tilt can also be used as an option to remedy this aspect but only extends reliably to a maximum 3–6° (Ishizuka, 1982 ▸; Chen *et al.*, 1997 ▸). Simulations with arbitrary orientations could also be performed by considering all unit cells in a crystal, while adding zero padding at its ends. Even though modelling a full crystal can quickly become intractable computationally, the use of a smooth envelope function may improve this commonly admitted limitation of MS (Kirkland, 2019 ▸). A few very efficient MS implementations have been reported to date (Ophus, 2017 ▸). Some of them are targeted at convergent-beam electron diffraction (CBED), while others have special features such as inclusion of inelastic scattering (Allen *et al.*, 2015 ▸). Yet, an approach combining full modelling capabilities with an acceptable computational efficiency, designed specifically for the continuous-rotation electron diffraction of large organic structures, does not seem to be available. Such an approach would indeed be of significant value for macromolecular structure determination from ED data.

On the other hand, the BW method can simulate ED for structures in arbitrary orientations but does not scale well with the structure size. Although a few rather efficient BW implementations (Zuo & Weickenmeier, 1995 ▸) have been proposed to include a large number of beams, BW can hardly be applied to very large structures since the scattering matrix becomes prohibitively large. Due to the unfavourable 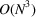 complexity of matrix diagonalization, where *N* is the number of beams, the method is hardly applicable in practice to the determination of macromolecular structures where it would have to be done numerous times. Non-periodic structures, defects and solvent scattering are also hard to model with this method.

In other areas of physics, specialized multiple scattering approaches have also been developed. In particular, the T-matrix approach has been extensively applied to wave propagation related problems such as electromagnetics (Hamid *et al.*, 1990*a* ▸,*b* ▸; Eremin *et al.*, 1995 ▸), optics (Moine & Stout, 2005 ▸) and acoustics (Silva *et al.*, 2012 ▸; Godin, 2011 ▸). Although it does not compete with MS from a computational standpoint, this approach benefits from providing an exact solution to Schrödinger’s equation for an ensemble of spherically symmetric atomic potentials in the independent atom model (IAM) approximation. Besides, *ab* *initio* real-space multiple scattering calculations have also been developed in X-ray photon emission spectroscopy with elaborate potentials including many-body effects (Rehr *et al.*, 2009 ▸). Similar approaches based on the T-matrix also exist in electron energy-loss spectroscopy (Sébilleau *et al.*, 2006 ▸).

### 1.3. Contribution and outline

The purpose of this paper is to adapt the T-matrix formalism specifically to the case of scattering of fast electrons by light-atom structures. In particular, we present an intuitive picture of multiple scattering in the forward scattering approximation. We propose a comparison with the MS interpretation of multiple scattering. In addition, we discuss the validity of the forward scattering approximation and the phase grating approximation used in the MS approach. Finally, we draw conclusions and outline extensions of the proposed approach in order to account for incoherent inelastic electron scattering.

## 2. Theory

The scattering of fast electrons by an atomic structure is described by the wavefunction Ψ, obeying the following Schrödinger’s equation (Kirkland, 2019 ▸): 

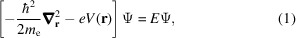

where 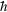 is Planck’s constant, 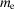 the mass of the electron, *e* the elementary charge, 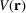 the spatially varying electrostatic potential created by the atoms, and *E* the electron energy.

In an ED experiment, 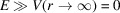 , which corresponds to the continuum, non-quantized state of the system (Chuang, 1996 ▸). Therefore, we solve (1) with *E* as a parameter, equal to the energy of incident electrons.

The total wavefunction Ψ is described as the sum of an incident wave 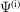 and a scattered wave 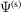 : 

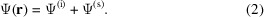

The incident wave is assumed to be known, *i.e.* a plane wave or a Gaussian wave for example. The T-matrix aims at determining the scattered wave 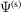 by the sample.

### 2.1. T-matrix formulation

In its standard form, the T-matrix approach solves for the case where the incident wave is described by a plane wave of wavenumber 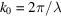 (optics convention), and the electrostatic potential is modelled by a uniform constant inside non-overlapping spheres. The constant potential and radius of the spheres depend on the atom type.

Although this is not an accurate representation of the actual potential profile 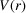 , using an appropriate amplitude for the constant potential 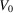 should provide similar values of scattering cross sections. The scattering cross section being a reliable figure of merit of the strength of an interaction, one can assume that a suitable choice of radius and constant potential should therefore provide a faithful depiction of the extent of multiple scattering in the structures of interest.

**Figure 1:**
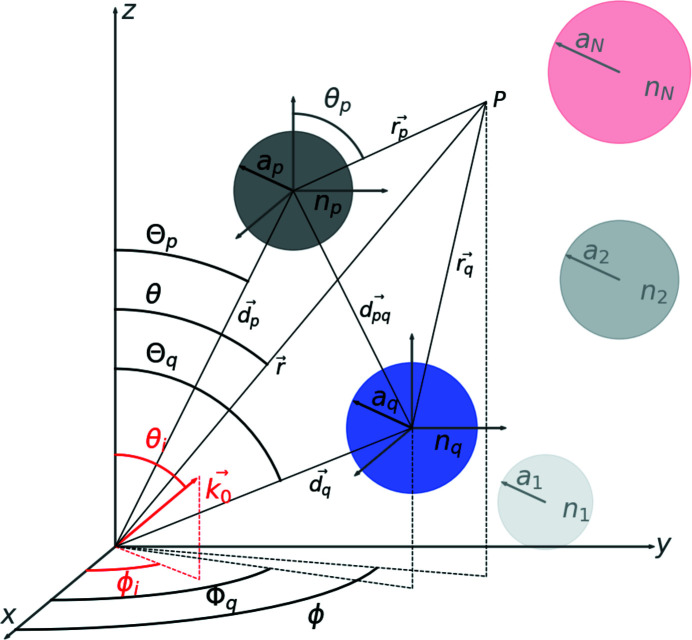
Setup and conventions for the scattering problem solved with the T-matrix approach. A plane wave with a wavevector **k**_0_ with incident angles ( 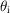 , 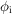 ) interacts with *N* spherical atoms, each with individual location (for example, atom *p* at **d**_*p*_), radius ( 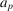 ) and electrostatic potential strength ( 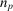 ). The scattered wave is observed at observation point P at **r** in the global reference frame and at individual distances in the local reference frame of the spheres (for example, 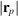 ). See discussion in the text for additional details. In practice, the incident plane wave is taken along the +*z* axis and the structure oriented accordingly.

The setup is shown in Fig. 1 ▸. This formulation is well established (Hamid *et al.*, 1990*a* ▸,*b* ▸; Eremin *et al.*, 1995 ▸) and the theory is outlined here for the purpose of introducing the forward multiple scattering approximation. In the T-matrix approach, the electrostatic potential is assumed constant inside different regions of the 3D space. In each region, the problem is reduced to the Helmholtz equation in spherical coordinates: 

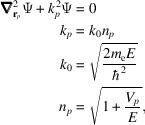

where 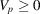 is the constant positive potential inside sphere *p* of radius 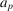 centred at 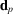 , 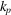 the wavenumber inside the sphere and 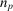 is sometimes referred to as the refractive index by analogy with optics. In the remainder, 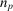 will be referred to as the potential strength for clarity purposes. The scattered wavefunction 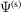 can be decomposed into the scattered wavefunctions of individual atom spheres, 

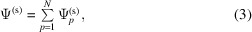

where *N* is the number of spheres. The *p*-sphere scattered wave 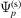 can itself be partitioned into a part inside sphere *p* noted 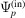 and a part outside sphere *p* noted 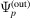 . The expressions for 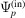 and 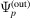 are detailed as

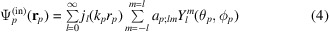




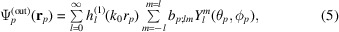

where 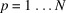 , 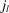 and 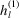 are the spherical Bessel and Hankel functions of the first kind, respectively, 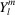 are the spherical harmonics of order *l* and azimuthal order *m*. Note that these equations are expressed in the reference frame of each sphere *p*, hence the use of variable 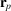 .

The unknown coefficients 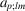 , 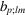 are found by imposing the continuity of the wavefunction and its radial derivative at the surface of each sphere *p*. After some mathematical manipulations (see Appendix *A* [App appa]), this results in the following linear system of equations: 

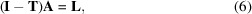

where **I** is the identity matrix, **A** the unknown vector of coefficients, **T** the cross-coupling matrix and **L** the known incident wave which appears on the right-hand side of (6).

Note that, in this formulation, the unknown coefficients, found from the continuity interface conditions, will model the response of each individual sphere to both the incident wave and the waves scattered by the other atoms. This model therefore does account for multiple scattering.

### 2.2. Far field and scattering cross section

In electron crystallography, diffraction patterns are recorded that correspond to the intensity of the scattering amplitude profile 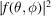 for various crystal orientations. A diffraction pattern is obtained in the far-field diffraction regime, which arises when the shape of the angular radiation scattering amplitude 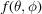 no longer depends on the distance to the scattering specimen. Using the asymptotic behaviour 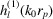 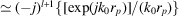 and since 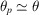 , 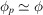 , the far-field scattering amplitude 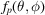 from each individual sphere *p* can be written as

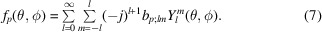

The total far-field scattering amplitude 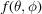 is the sum of the scattered field contributions from all individual spheres. Note that the scattered field from each individual sphere already accounts for the scattering between the spheres through the coefficients 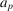 and 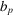 . Since, in the far field, 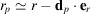 ,

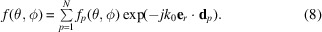

The normalized differential scattering cross section can be obtained as

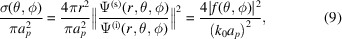

where we have used 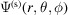 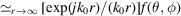 .

## 3. Real-space forward multiple scattering picture

### 3.1. T-matrix forward scattering approximations

Equation (6) is a convenient way to represent the system as it readily identifies **L** as the solution to the uncoupled (**T** = 0) problem. If **T** = 0, the system can be broken down separately into the scattering problems for each individual sphere, for which the solutions are immediately identified as 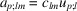 , 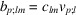 . These are indeed the well known analytical solutions of Mie scattering by a soft sphere (Balanis, 1989 ▸).

The cross-coupling matrix **T** accounts for multiple scattering effects. If **A** is written as 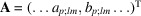 , then **T** is a matrix with only off-diagonal components: 

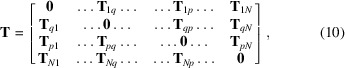

where **T**_*pq*_ represents the scattering from sphere *p* due to the scattering from sphere *q*. By considering all components 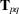 , 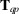 , we assume that the scattering from sphere *p* affects scattering from sphere *q* and vice versa. Matrix (10) is completely filled with nonzero entries apart from its diagonal components. It is therefore not a sparse matrix, which implies quadratically growing memory requirements. Moreover, since full intersphere scattering is considered, the solution of equation (6) requires matrix inversion which is computationally expensive.

However, as detailed below in Section 4.2, backscattering can be neglected for very fast electrons, which is known as the forward scattering approximation. This results in **T** being lower triangular if the spheres are sorted in ascending order along 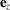 . In the case of atoms lying in the same coordinate plane, *i.e.* slice, the atoms can be sorted according to their transverse coordinates. Then, as 90° scattering is neglected in the forward scattering approximation, the T-matrix remains lower triangular. Inversion of the lower triangular matrix is computationally tractable and calculations can be performed sequentially one slice after the other. Therefore, the forward scattering approximation converts the implicit self-consistent T-matrix scheme into a forward scattering explicit scheme similar to the approach taken in the MS method.

### 3.2. Multiple scattering approximations

**Figure 2:**
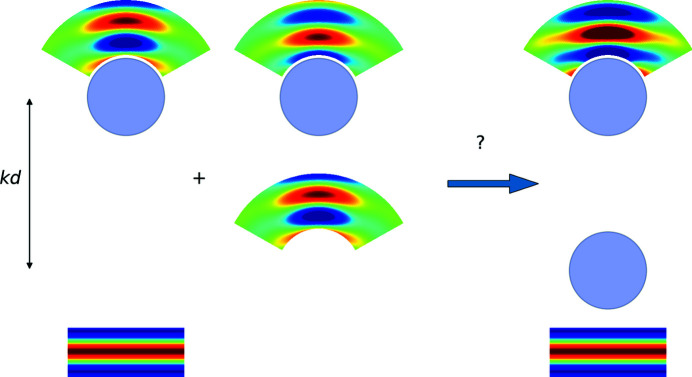
Two-level forward scattering approximation using the T-matrix approach with an incident plane wave propagating upwards. The third column represents scattering from the top scatterer, located at distance *kd* from the bottom scatterer. By neglecting backscattering, this can be approximated by the sum of (i) the kinematic scattering of the incident plane wave by the top scatterer (first column) and (ii) the secondary scattering following kinematic scattering from the bottom scatterer (second column). The scattering from the bottom scatterer is viewed by the top scatterer as a particular incident wave (neither planar nor spherical) emitted from a point source at a distance *kd*.

Since 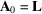 represents single scattering, we can establish that 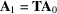 accounts for secondary scattering. Similarly, outward scattering amplitudes from sphere *p* can be written as

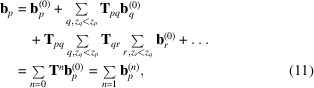

where the first term accounts for kinematic scattering, the second term for secondary scattering, the third term for tertiary scattering, and so on. This is a development similar to the Korringa–Kohn–Rostoker (KKR) theory of multiple scattering (Korringa, 1947 ▸, 1994 ▸; Kohn & Rostoker, 1954 ▸). This forward multiple scattering picture is illustrated in Fig. 2 ▸ for the case of a two-atom system. From a computational point of view, equation (11) may offer an advantage over the full forward scattering approximation if only a few *n*-time scattering terms are necessary. Indeed, since each term in the expansion depends on precomputed coefficients 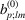 , computation of the 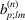 coefficients for the *n*th scattering term can be massively parallelized for each atom.

### 3.3. Scattering probabilities

The probability of an electron being scattered elastically *n* times can be estimated classically from ballistic arguments using a continuous model of matter. It can be established (Egerton, 2011 ▸) that the probability of an electron being elastically scattered *n* times after passing through an amorphous sample of thickness *z* follows a Poisson distribution 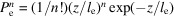 , where 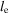 is the elastic mean free path. It is paramount to note that this classical expression models incoherent scattering, not including interference effects between multiple scattering events. This model is therefore valid for classical particles not exhibiting particle–wave duality behaviour. In quantum systems, it is also possible to consider incoherent scattering if inelastic scattering is strongly present. Indeed, inelastic scattering not only induces a transfer of energy between scatterers but also a loss of coherency through phase shifts (Howie, 2014 ▸). As a result, quantum systems, where inelastic scattering is predominant, would scatter incoherently. For example, inelastic scattering may be due to electron–electron interactions, solvent or impurity scattering, as well as thermal diffuse scattering.

In this section, we suggest a way to consider probabilities of multiple coherent elastic scattering events. It will be shown that while multiple scattering amplitudes can be considered, it may not be possible to identify corresponding probabilities without any ambiguity precisely because of interference.

Using a wave-like description, the probability of an electron undergoing a scattering event can be determined from the scattering cross section as follows. Let *S* be the area over which the specimen is illuminated.

Let *z* be the thickness of the specimen. The incident electron plane wave can be normalized by 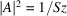 so that it integrates over the interaction volume 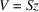 to a single electron. Then, the flow of electrons per unit time per unit area (Vainshtein, 1964 ▸) is defined as 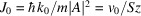 , where 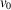 is the speed of the incident electrons.

By definition of the cross section, 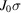 is the probability of an electron being scattered every second. Since it takes a time 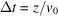 for an electron to pass through the specimen, the overall probability of a single electron being scattered is therefore 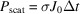 . Therefore, after inserting the expressions for 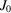 and 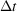 ,

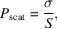

where *S* can for example be the area of the aperture in selected-area electron diffraction (SAED). When performing a MS simulation, *S* is the area of the simulated domain (transverse supercell if performing a periodic simulation).

Here, we define 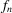 as the complex scattering amplitudes obtained by considering scattering from the wave being scattered *n* times. It can readily be established that

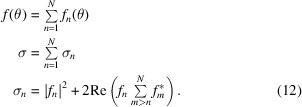

We can then define 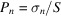 as the probability of an electron being scattered *n* times. The choice of *n*-time scattering cross section 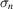 is arbitrary precisely because of the cross-coupling interference terms 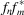 . It has been chosen in such a way that when more *n*-scattering terms are taken into account, probabilities of higher *n*-level scattering events are transferred from the lower *n*-level scattering events. In the case of an application to two-beam scattering theory, the intensity of the forward and scattered beams oscillates as the thickness of the sample grows. The scattered beam is zero when the forward beam has been scattered an even number of times and maximum when the forward beam has been scattered an odd number of times (Cowley & Moodie, 1957 ▸). Our definition of scattering probabilities should reflect this behaviour.

The probability of an electron not being scattered is naturally 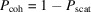 , where 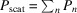 . Note that, since 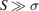 , the probability of scattering is necessarily less than 1.

For a very large number of scatterers, *N*, regularly spaced by distance *dz*, we can define the average cross section 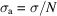 . The corresponding scattering probability is then 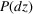 = 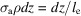 where 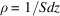 is the density (since *Sdz* contains only one scatterer) and 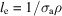 is the mean free path. This is consistent with the definition used by Latychevskaia & Abrahams (2019 ▸).

### 3.4. First Born approximation and kinematic scattering

Let us assume normal incidence with 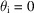 , so that 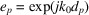 , and note that the Ewald sphere can be represented by **q** = 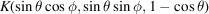 in reciprocal space (or transfer vector momentum space), with 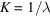 being the wavenumber in crystallographic convention. Keeping only the kinematic term in equation (11) and inserting in the far field in equation (8) give

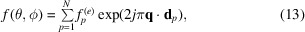

where 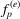 is the scattering amplitude of sphere *p* in the first Born approximation, also known as the form factor. Equation (13) corresponds to the standard kinematic formula for the structure factor traditionally used in crystallography. Note that the kinematic approximation is nothing else than the first Born approximation applied to the whole assembly of atoms.

It will be shown in Section 4.2 that the first Born approximation holds in the case of single scatterers, but fails in application to multi-atom systems. This means that the kinematic approximation is not generally valid.

### 3.5. Multiple scattering in the MS approach

In MS, the forward scattering approximation is used and the potential discretized in slices. The wavefunction is propagated from one slice to the other using a Fresnel propagator.

A multiple scattering interpretation of the MS approach has been proposed (Cowley & Moodie, 1957 ▸), which, although analogous to the one presented above, differs in that it is stated in reciprocal space. More details on the differences between the two interpretations can be found in Appendix *B* [App appb].

## 4. Application and results

Very efficient open-source packages exist for T-matrix calculations, but they are targeted at applications in electromagnetics (Egel *et al.*, 2017 ▸; Kottke, 2020 ▸). There are also packages targeted at photon and electron spectroscopy and related to the T-matrix, such as *msSpec* (Sébilleau *et al.*, 2011 ▸) and *FEFF9* (Rehr *et al.*, 2010 ▸). Here, an open-source package, adapted to high-energy electron diffraction and including the forward scattering approximation, has been developed and made available (Drevon, 2021 ▸).

### 4.1. Validity of the implementation

In practice, the size of the matrix in equation (6) has to be truncated to a maximum integer order 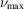 due to computer memory limitations.

A rule for obtaining accurate results is to use an integer larger than the maximum value of the normalized radius *ka*, which we call 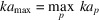 where 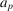 is the radius of the spheres and *k* the wavenumber.

**Figure 3:**
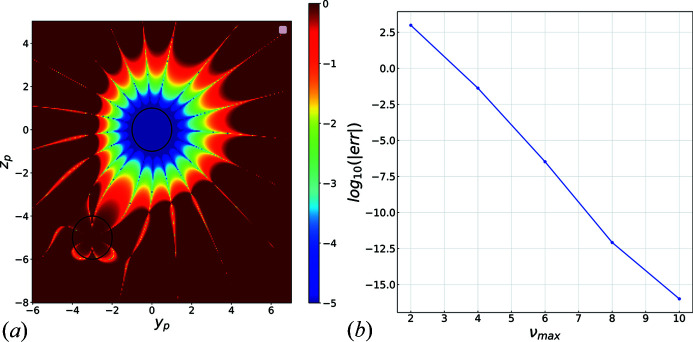
(*a*) Error difference in evaluating 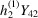 using (i) the standard product of the radial Hankel function 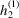 and spherical harmonic 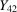 and (ii) the translational addition theorem (14), (15) at a distance 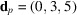 (in normalized radii) from the origin with expansion order 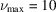 . The circle represents spheres of radius 1. Colour axis in log scale. (*b*) Continuity error at the sphere boundaries [*i.e.* difference between the solution computed at the boundary using equations (4) and (5), integrated over the sphere boundary]. For this example, 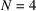 and 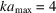 . The error reaches machine accuracy at expansion order 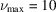 .

Besides, the translational addition theorem (Dufva *et al.*, 2008 ▸) used for expressing the scattered field of any sphere in the reference frame of another sphere introduces an approximation of the translated spherical Hankel functions. This theorem is also more commonly known as the structure constant expansion or Kasterin expansion. The computational accuracy of this translation decreases from the centre at which this expansion is written. This is analogous to a Taylor expansion, for which the accuracy away from the point at which it is expressed increases with increasing expansion order. Note that the distances between the spheres do not affect the accuracy of this translation operation and should not affect the choice of 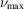 . Once again, the radius of the spheres determines the choice of 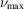 . This is illustrated in Fig. 3 ▸(*a*), where the error between 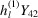 , computed at the origin, using the translational addition theorem with 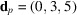 and 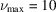 , is displayed in log scale.

The choice of 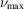 is best evaluated by assessing the continuity of the wavefunction at the surface of the spheres as shown in Fig. 3 ▸(*b*) for 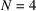 and 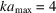 . This is also used to validate the correctness of the implementation since it can be seen that machine accuracy can be reached when increasing the order of the expansion.

### 4.2. Validity of forward scattering and phase grating approximations for light atoms

In the case of very fast electrons typically used in transmission electron microscopes, *E* = 50–300 keV. Inclusion of relativistic effects results in a wavelength λ = 0.02508 Å at 200 keV, which will be assumed from now on unless stated otherwise.

**Figure 4:**
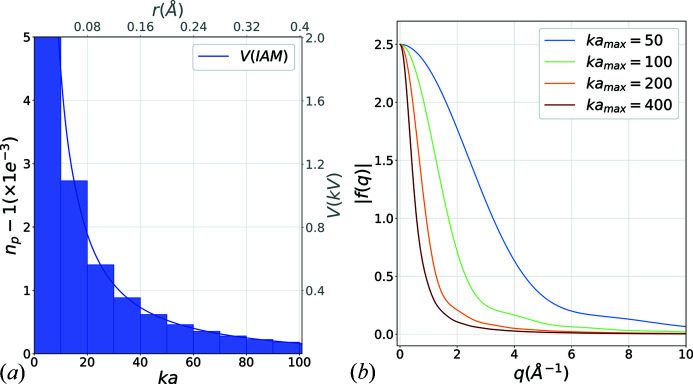
(*a*) Electrostatic potential, created by an atom of carbon in the IAM (blue solid line). The blue patches show a multi-shell approximation model, which can be used to represent the potential in a T-matrix approach. (*b*) Scattering amplitude in the Born approximation for the multi-shell model with increasing truncation radius 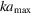 .

In the IAM approximation, the Coulomb potential is created by the charge of the nucleus and its electron cloud. It is typically fitted with a sum of three screened Coulomb potentials and three Gaussian terms (Kirkland, 2019 ▸) which results in the potential shown in Fig. 4 ▸(*a*).

The solution to Schrödinger’s equation in such a potential can only be solved perturbatively (Müller, 1965 ▸) and is beyond the scope of this study. However, indicative values for 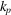 and 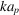 can be used with a multi-shell representation as shown in the form of blue patches in Fig. 4 ▸(*a*). Although the range of the screened Coulomb potential is theoretically infinite, it can be truncated to radius *ka* for most practical purposes. This Coulomb potential truncation is commonly known as the muffin-tin model. The number of concentric spheres to use has mainly an effect on the large-angle representation of the form factor which does not impose severe constraints in a low-angle forward scattering approximation. In Fig. 4 ▸(*b*), the multi-shell scattering amplitudes calculated in the Born approximation are shown for increasing values of truncation radius. The figure only shows a satisfactory agreement with the electron diffraction scattering factors for normalized radius as large as 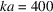 . Even though the potential 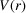 is extremely small at such a large value of the radius 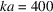 , those shells are required to account for the proper low-angle representation of the form factor.

**Figure 5:**
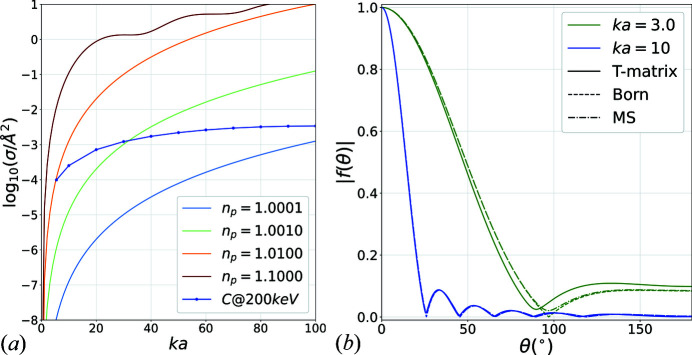
(*a*) Total scattering cross section σ at 200 keV for a few values of 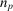 over a range of normalized radii *ka*. The dark blue curve shows the location of the carbon spherical shells. (*b*) Shape of the scattering amplitude for normalized radius. The green curves correspond to 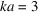 and potential’s strength 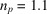 . The blue curves correspond to 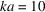 , 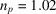 which is one of the carbon shells. Dashed lines show the Born approximation and dashed–dotted lines show the phase grating approximation used in MS. The Born approximation is very good for the carbon data point used.

As mentioned above, ED simulations based on the T-matrix approach are very expensive computationally at 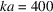 , because higher-order terms of series in equations (4), (5) would need to be included. However, the medium truncation range should be sufficient for providing a reasonable picture of dynamical scattering. Fig. 5 ▸(*a*) shows the total scattering cross section of a single sphere with increasing radius for a range of values of potential strength 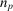 . The dark blue curve shows the locations of the spherical shell for carbon. It becomes almost flat for the parameter set ( 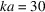 , 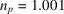 ), from which it keeps increasing very slightly to reach the asymptotic value 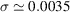 . This value is almost identical to the average elastic cross section for real carbon in the Born approximation (Latychevskaia & Abrahams, 2019 ▸) which confirms that the chosen parameters capture the strength of incident electron–carbon interaction. Therefore, even though the chosen parameter set is a coarse representation of the potential profile of a real carbon atom, it should provide a reasonably faithful estimate of the extent of multiple scattering of real carbon-based samples, which is the main objective of this study.

Fig. 5 ▸(*b*) shows the shape of the far-field amplitudes for two sets of *ka*, 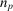 values. The first one is for the extreme value of 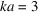 and 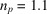 while the second corresponds to one of the carbon atoms. While differences are obvious for the first case, the Born approximation is very accurate for the carbon atoms, although some minor differences appear at low angles where the phase grating approximation provides some improvements too.

**Figure 6:**
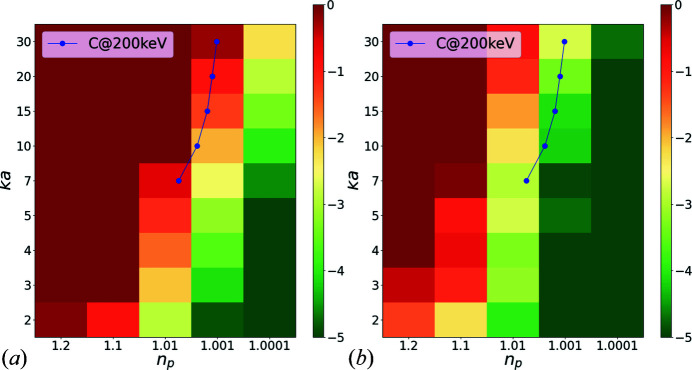
Error of 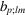 [equation (14)] in 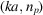 space (colour axis in log scale) for a two-scatterer system using the (*a*) kinematic scattering approximation, (*b*) forward scattering approximation. The blue dots correspond to the location of the spherical shells of a carbon atom at *E* = 200 keV (shown only for first five shells for visualization purposes).

Fig. 6 ▸(*a*) shows a 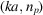 map of the difference between the exact coefficients of the two-body problem and those calculated using the kinematic approximation. This error is calculated as

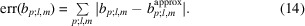

Fig. 6 ▸(*b*) shows the same thing as a result of using the forward approximations.

For this case, a value of 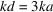 is used, which is close to interatomic distances. Overall, it is clear that the forward scattering approximation is very accurate over all ranges of parameter sets for carbon atoms, which is expected since there is very little backscattering beyond 90°. On the other hand, the kinematic approximation does not appear quite as good, even for this, a mere two-atom problem. Although not shown here, both the kinematic and forward scattering approximations tend to work slightly better with increasing distances *kd*, since coupling between the spheres gets reduced and, therefore, is less likely to affect scattering from the other spheres. Using low values of 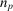 results in an overall good approximation of both the uncoupled and forward scattering approximation. This is an anticipated result, since for weak potentials the kinematic approximation works better. The uncoupled approximation improves with decreasing radii, since a small *ka* results in a small scattering cross section. On the other hand, the forward scattering approximation improves with larger values of *ka*, which make backward scattering less likely.

**Figure 7:**
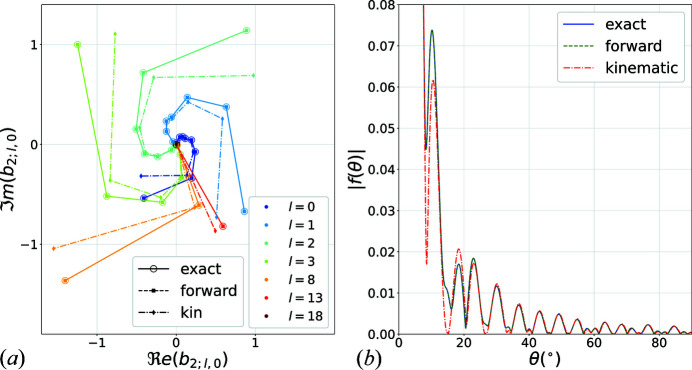
(*a*) Complex space scattering coefficients 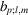 for a two-body problem for 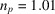 , 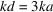 with increasing radius *ka*. Only the coefficients for sphere 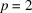 and 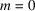 are shown. The coefficients were computed from full T-matrix (open circles), in the forward scattering approximation (squares) and the kinematic approximation (diamonds). (*b*) Comparison of far-field scattering amplitudes for 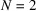 , 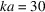 , 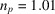 , 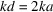 using full T-matrix (solid blue), in forward approximation (green dashed line) and kinematic approximation (red dashed–dotted). The forward approximation is indistinguishable from the exact solution in this case.

Fig. 7 ▸(*a*) shows the scattering coefficients 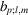 in complex space for the given two-body problem, where 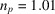 , 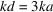 , while increasing radius *ka* (up to 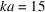 ) for a selected set of orders *l*. Only the coefficients for sphere 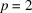 are shown since it is the sphere most affected by the approximations. Here, only 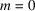 coefficients are used, because in the case of planar illumination, this configuration has azimuthal symmetry. The coefficients are computed using the full T-matrix, the forward scattering approximation and the kinematic approximation. In order to get this picture, the coefficients were divided by the phase factor at sphere *p*, *i.e.* 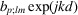 . The arms of the spiral are rotated by 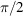 from one order to the other as a result of the 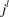 factor in the spherical expansion of the incident plane wave. All the coefficients increase with increasing *ka* as a result of the increasing scattering cross section with increasing *ka*. For a given *ka*, the strongest contributing term orders are around 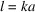 as a direct manifestation of the convergence behaviour presented above. It can be observed that the kinematic approximation error increases with the radius while the forward approximation is very accurate across all radii.

Fig. 7 ▸(*b*) shows a comparison of the computed far-field scattering amplitudes for 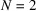 , 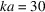 , 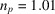 , 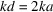 using the full T-matrix, the forward approximation and the kinematic approximation. The peaks and valleys are both due to the single scattering profile of the constant sphere and the interferometric path length 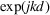 . It is possible to see that the coupled problem has a slight averaging effect over the kinematic pattern, a well known feature of dynamical diffraction.

### 4.3. Successive multiple scattering approximations

Finally, we consider an array of *N* identical scatterers regularly spaced by 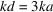 which correlates with lengths in molecules.

Here, we consider the successive multiple scattering approach under normal illumination. This is an extreme case where strong dynamical scattering is expected.

**Figure 8:**
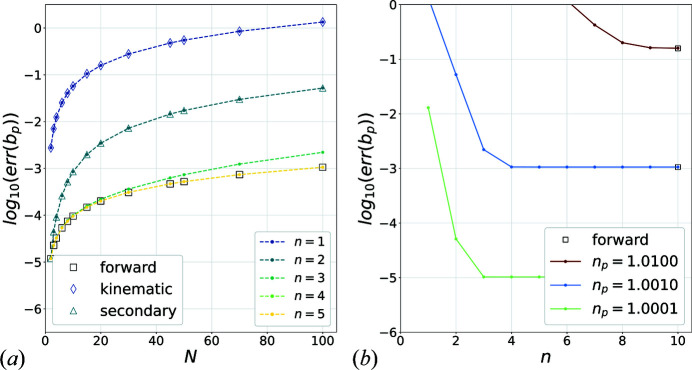
(*a*) Error [equation (14)] on the scattering amplitude coefficients with increasing number of spheres *N* for 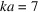 , 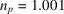 , 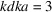 . Squares: forward scattering approximation; diamonds: kinematic approximation; triangles: secondary approximations; coloured dashed lines: successive *n*-times multiple scattering approximations. (*b*) Error on the scattering amplitude coefficients in the increasing successive approximations for 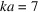 , 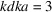 and 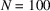 spheres while varying 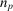 .

Figs. 8 ▸(*a*) and 8 ▸(*b*) show the evolution of the error [equation (14)] on scattering amplitude coefficients for an increasing number of spheres while also varying the number of successive approximations while keeping parameters 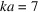 , 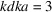 fixed.

The forward scattering approximation becomes worse for increasing numbers of spheres as a result of accumulated errors. This is a limitation of a forward propagation scheme such as the one used in the MS approach, whose accuracy gets worse with increasing simulated sample thickness (Kirkland, 2019 ▸). Overall, the successive approximation schemes naturally converge to the forward approximation for sufficiently large *n*. However, higher-order terms need to be included in equations (4), (5) as the number of spheres (or thickness of the sample) increases. This result is similar to other multiple scattering approaches. For example, in the case of a two-beam configuration, it was shown (Cowley & Moodie, 1957 ▸) that the scattered beam could be expressed using expansion (17), considering only the scattering terms of the primary beam 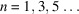 . The famous two-beam analytical scattering expression is obtained as the sum of the infinite series.

**Figure 9:**
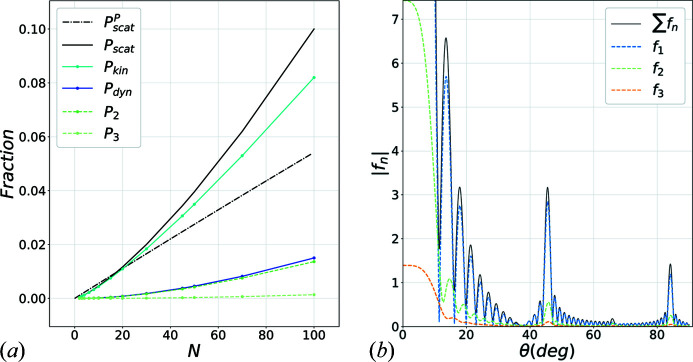
(*a*) Scattering probabilities (dashed coloured lines) of *n*-times scattering, kinematic (cyan solid line), dynamic scattering (blue solid line) and total scattering (black solid) probabilities. The total scattering probability following the Poisson distribution using the average scattering cross section is shown as the black dashed–dotted line. Probabilities have been integrated over angular distribution using 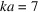 , 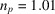 and 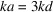 . (*b*) Scattering amplitudes 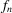 , associated with *n*-times scattering (for *n* = 1-, 2- and 3-level scattering) for 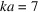 , 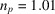 and 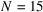 . The total scattering amplitude 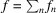 is shown in solid black.

Fig. 9 ▸(*a*) shows the scattering probabilities of *n*-times scattering for 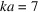 , 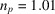 , 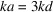 with increasing *N*. The dynamic scattering probability 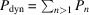 and the kinematic as well as the total scattering probabilities are shown. It seems that all multiple scattering terms increase with the number of spheres, in contrast to the ballistic picture of the Poisson distribution (Egerton, 2011 ▸), also shown as dashed–dotted lines calculated using the average scattering cross section. The main reason for these trends is that the ballistic picture ignores the effect of interference, which drastically affects the overall scattering process.

Fig. 9 ▸(*b*) shows the scattering amplitudes 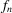 , associated with *n*-times scattering for 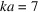 , 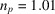 and 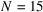 . The scattering amplitudes have similar shapes, which mostly contribute to constructive interference, although slight peak misalignments can be seen. The reason for the large-angle scattering, visible on the figure, is due to the small radius used in this example. As mentioned above, a much larger normalized radius would be required to properly account for the low-angle representation of the far-field diffraction pattern. However, because of the comparable scattering cross section of the chosen model and a real carbon atom, the extent of multiple scattering should have been reasonably estimated.

Note that the examples presented here have been performed for electron incident energies of 200 keV, which is a typical value in transmission electron microscopy. Decreasing the energy would result in lower normalized radii, which would correspond to shifting the blue curve for carbon in Fig. 5 ▸(*a*) to the left. As a result, spherical harmonics of lower orders would be sufficient to achieve good accuracy. On the other hand, the forward scattering approximation would lose accuracy at smaller thicknesses, and then full inversion of the system (6) might be required. For higher energies, the forward approximation becomes more accurate but the size of the system increases, making the problem computationally harder.

## 5. Conclusion and perspective

An alternative approach, based on the T-matrix formalism, has been applied to the scattering of fast electrons, by light-atom structures. The validity of important approximations, used in the MS approach, has been discussed, and a multiple scattering approximation framework has been proposed and put in perspective compared with other existing interpretations. While it is worth mentioning that the T-matrix does not scale favourably with large increasing values of the wavelength-normalized radii *ka*, as commonly used for high-energy electron diffraction, it can still provide valuable insights in understanding dynamical diffraction effects thanks to its ability to solve the wave equation exactly. For a 200 keV electron beam, each spherical coefficient 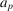 , 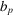 accounts for one wavelength of physical space comparable with the sampling resolution used in typical MS simulations. For a truncation longitudinal number 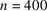 , the total number of coefficients for all atoms would be 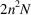 since azimuthal coefficients 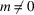 are necessary for nonlinear array arrangements. For *N* = 10 000 atoms, this is on the order of a few gigabytes of memory. While inverting a matrix of this size would be computationally intractable, the forward scheme approximation, presented in this paper, makes such a computation viable. Indeed, it offers the possibility of parallel computation for all atoms in the same slice, similar to the way it is traditionally done in finite difference schemes.

In the presented approach, the spherically symmetric effective potential does not model the real electrostatic potential very accurately. However, we anticipate that the proposed interpretation of multiple scattering is equally applicable to the more accurate case of a screened Coulomb potential. Such a potential could naturally be included by using a basis of radial functions representing solutions to the homogeneous Schrödinger’s equation, which can be obtained numerically. In fact, since the T-matrix approach mainly relies on the spherical symmetry of the individual scatterers, it can be adapted to any family of radial functions, provided that translational coefficients (15), (16) can be computed numerically.

For periodic structures, the Green’s functions (GFs) could be used as basis functions. All GFs are solutions to Schrodinger’s equation in a periodic potential, expressed as an expansion upon the lattice vectors of the crystal under consideration. They would automatically satisfy the boundary conditions imposed by the Bloch theorem in crystals. This is the starting point of the Kohn–Rostoker method (Kohn & Rostoker, 1954 ▸).

An apparent limitation of both T-matrix and MS approaches lies in the use of the IAM, which, by definition, ignores the effect of bonding. Theoretically, such bonding could be included (Natoli *et al.*, 1986 ▸). In fact, the MS approach can be adapted, at a computational cost, to bonding models such as the transferable aspherical atom model (TAAM) or even potentials determined by density functional theory. On the other hand, the single-site T-matrix approach, presented in this paper, cannot work directly with such potentials because the spheres are treated as non-overlapping. A possible option would be to employ effective potentials calculated in the framework of self-consistent electronic structures, where the effect of bonding manifests itself in the bonding-corrected effective potential. However, it is still an open question whether the effects of such bonding play an important role in structure determination of organic structures by electron diffraction.

The advantage of the multiple scattering approximation is that it may offer the possibility to include consistently the effect of incoherent and inelastic scattering, which can provide a greater insight into dynamical diffraction effects in real experiments. Incoherent scattering could, for example, be included in a stochastic fashion as randomly affecting the phase of the scattered waves. Inelastic scattering might require the use of dedicated electrostatic absorption potentials. It is anticipated that incoherent and inelastic scattering may have a significant effect in dynamical diffraction even when energy filters are used.

## 6. Data availability statement

Computer codes, developed in this study, are freely available under the GNU GPL licence v3.0 from https://github.com/ronandrevon/pyScatSpheres. No new ED data were produced in this study.

## Figures and Tables

**Figure 10:**
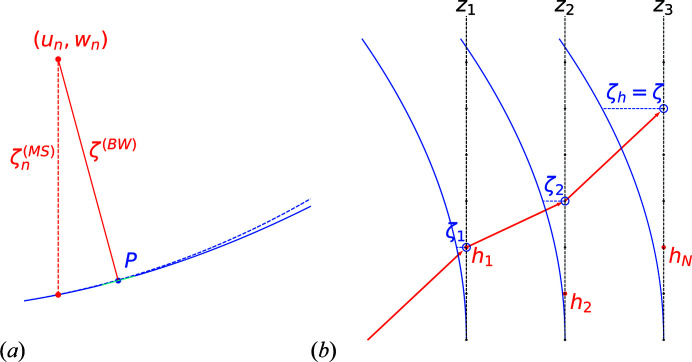
Multiple scattering in MS. (*a*) Distance 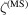 to the Ewald paraboloid (solid curve) as represented by MS and distance 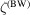 to the Ewald sphere (dashed curve), known as the excitation error in the Bloch wave theory. Point P is the projection of reciprocal point 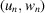 onto the Ewald paraboloid. (*b*) Multiple scattering in reciprocal space for 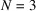 slices located at 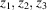 . In this example, the beam is scattered in the first slice at 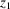 in direction 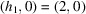 . Then, at slice 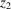 , it scatters in direction 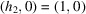 , which results in an overall scattering of the original beam in 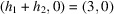 . Finally, at the last slice 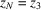 , deflection is in direction 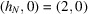 . Therefore, the overall scattering for this example corresponds to a contribution to reflection 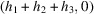 = (5,0) in the diffraction pattern. The open blue circles show how to interpret the successive excitation errors 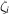 .

## Appendix A T-matrix

The unknown coefficients  ,  are found by imposing the continuity of the wavefunction and its radial derivative at the surface of each sphere *p*:

where  is the incident electron wavefunction,  the scattered wavefunction of sphere *p* expressed inside the sphere,  the scattered wavefunction of sphere *p* expressed outside sphere *p*, and  the radius of sphere *p*.

Using the orthogonality of the spherical harmonics, the following linear system yields the unknown coefficients:

where the translational addition theorem (Dufva *et al.*, 2008 ▸) has been used to express the field, scattered by sphere *q* in the reference frame of sphere *p*, formally written as  . This operation involves the coupling coefficients  , where  .

The coefficients  are related to the incident wave. In the case of a plane wave  ,  , the addition theorem is used to expand the plane wave on the spherical Bessel solutions basis set:

where  are the spherical coordinates of the centre of sphere *p* in the global coordinate system and  is the angle of incidence with respect to the  axis. For propagation in the  plane, assumed in this paper,  and  is the phase offset at sphere *p*. The different notations are illustrated in Fig. 1 ▸.

The coefficients  and  are expressed as

where  .

Each of equations (15), (16) represent  equations for individual spheres where  is the truncation longitudinal order. Note that each equation involves the outside coefficients  of all the other spheres through the translational coefficients. As a result, equations (15), (16) represent a  system with as many unknowns as the number of equations and, therefore, can be written in the matrix form (6).

## Appendix B Multiple scattering picture of the MS approach

The expression for the scattering amplitude  of beam  is proportional to the following expression:

where  ,  ,  ,  ,  ,  is the total thickness of the sample, *N* the number of slices of thickness  ,  =  +  ,  is the structure factor,  the interaction parameter, ζ the excitation error of beam  and  is the excitation error of beam  . The excitation error is expressed as

where *a*, *b* and *c* are the lattice constants of the crystal and  the wavenumber. Equation (18) expresses the longitudinal distance of beam  to the paraboloid shown in Fig. 10 ▸(*a*). This paraboloid is a very accurate representation of the Ewald sphere for large wavenumber *K*. Therefore, ζ is very close to the excitation error commonly defined in crystallography.

From equation (17) it is possible to gather terms in powers of  into  , which corresponds to the incident beam scattered *n* times before contributing to reflections  . The term  only appears for  and corresponds to the unscattered direct beam. There are *N* terms involving the factor  depending on which slice the single scattering event took place. There are  terms involving the factor  depending on which pair of slices the two-level scattering process is considered, and so on for the higher-order multiple scattering terms (Cowley & Moodie, 1957 ▸). This multiple scattering picture is illustrated in 2D in Fig. 10 ▸(*b*) for a case with  and using only beams in the zero-order Laue zone (ZOLZ)  .

Using only ZOLZ beams,  ,  is expanded as

which is the well known kinematic scattering regime, where the sum converges to the standard Ewald sphere curvature factor  for infinitely thick slices  ,  ,  .

For a two-level scattering using only ZOLZ beams,  would expand as (written in 2D here)
